# A Critical Review of Smart Residential Environments for Older Adults With a Focus on Pleasurable Experience

**DOI:** 10.3389/fpsyg.2019.03080

**Published:** 2020-01-24

**Authors:** Li Na Lee, Mi Jeong Kim

**Affiliations:** ^1^Department of Software and Information Systems, University of North Carolina at Charlotte, Charlotte, NC, United States; ^2^School of Architecture, Hanyang University, Seoul, South Korea

**Keywords:** smart environment, smart home, aging in place, older adults, pleasurable experience

## Abstract

Various smart services and technologies have been developed to support older adults' well-ness, make their daily tasks easier, and enhance their overall quality of life. When people grow older, older adults inevitably experience a significant decrease in their physical, cognitive, and sensory capabilities, which makes them develop negative attitudes toward technology. In this regard, this study highlights that older adults require not only usable and practical spaces but also smart residential environments that can fulfill them emotionally. Research on smart environments for this population should consider the hedonic and experiential factors of interacting with technology, such as fun, fulfillment, play, and user engagement. This study aims to provide a comprehensive review of smart residential environments to support positive aging and pleasurable user experience in the architecture domain. For this critical review emphasizing the pleasurable smart environment, an evaluation framework was developed, consisting of four categories: well-ness, independence, acceptance, and design. Through an extensive analysis of selected papers in the architecture domain, it was found that studies on the smart home tend to focus on utilitarian factors, such as usability, monitoring physical experiences, and simulating energy efficiency, and rarely mention psychological well-ness. Smart environments should be designed to not only emphasize efficiency, effectiveness, and satisfaction but also to engage older adults and provide them positive experiences. As various smart technologies continue to evolve and integrate into smart living spaces, it is important to understand older adults' cognitive and emotional aspects and make the smart environment a more comfortable place for them.

## Introduction

A “silver tsunami” is on its way. Silver tsunami refers to the rapid aging of the population and, in particular, of the baby boomer generation. This demographic shift has moved the focus of researchers, designers, health care providers, and policymakers from ascertaining ways to extend the lifespan to ways to improve the quality of life. The aging population will face attitudinal, environmental, and institutional changes in their later life (Bartels and Naslund, 2013). Living arrangements are dynamic because of changes associated with life events, such as widowhood, retirement, and changes in living environment. Age-related changes prevent older adults from actively participating in activities that are essential for their well-being. Owing to the rapid increase in the number of older adults, numerous smart technologies are designed to promote their well-being—including various health applications to support physical activities as well as sensor-based networks for monitoring activity—and to help these individuals stay in touch with family and friends (Morris et al., 2013; Yang et al., 2013; Lloret et al., 2015; Liu et al., 2016). Further, various studies are being conducted on the positive impact of smart technology on living environments, routine life activities, and maximizing the independence of older adults (Horgas and Abowd, 2004).

The concept of smart home has developed rapidly since the early 1990s, and various studies have been conducted in this research field. The smart home (also termed the intelligent home, aware home, and adaptive house) is a residence equipped with technologies that include sensors, wired and wireless networks, and intelligent systems (Bitterman and Shach-Pinsly, 2015). Over the past decade, smart home technology has increasingly targeted people having reduced capabilities due to age or disability. It was initially focused on increased security and energy savings, whereas, recently, the scope of its use has gradually varied to enhance the overall quality of life. However, the application and use of such technology in living spaces is limited. Moreover, few studies have considered older people's understanding of challenges or barriers that may occur in smart living and their acceptance of this concept. Their acceptance of assistive home-based technology is dependent on the complex relationships between cognitive and emotional components (Lê et al., 2012). We believe the aspects considered in designing smart residential environments for older adults in later life should include engagement and positive affect. Smart residential environments for older adults should be designed not only with an emphasis on providing efficiency, effectiveness, and satisfaction but also on engaging them and providing positive experiences. In this study, we focus exclusively on the living environment for older adults to promote pleasurable experiences. A pleasurable experience is defined in this study as one that allows aging occupants to have considerable amount of fun while living in a smart home and interacting with a variety of smart devices.

A variety of studies on the smart environment for older adults have been published in research disciplines, such as electrical engineering, information technology (IT), computer science, gerontology, biomedicine, and robotics, since the early 1990s (Bitterman and Shach-Pinsly, 2015). Although the smart environment involves the space in which human beings reside, the research areas of architecture and town planning have been less involved with smart home research. Research on the smart environment in the architecture domain tends to focus on utilitarian factors, such as ensuring usability, monitoring physical experiences, and increasing energy efficiency, and rarely considers users' emotional well-ness. The question is no longer only whether smart technologies are efficient, effective, or usable but how well these are able to engage older adults and provide them a positive and pleasurable experience as regards smart living. A smart environment in which older adults can experience emotional pleasure is required. To make smart homes more acceptable, by turning resistance into appreciation and providing pleasurable and positive experiences, technological understanding should entail spatial recognition. This study critically reviews the literature on smart environments that were published in the architecture research domain, using an evaluation framework consisting of four categories: well-ness, independence, acceptance, and design. This evaluation framework of smart environments for older adults has enabled a critical review of the selected papers. A Harvey Ball has been used to analyze the value of the smart environment of each paper based on the four categories indicated in the evaluation framework. As various smart technologies continue to evolve and integrate into smart living spaces, it is important to understand older people's cognitive and emotional aspects and make the smart environment a more comfortable place for them.

## Pleasurable Smart Residential Environments for Older Adults

Various types of living environments are available for older adults. Most older adults prefer to live independently in a familiar home setting as long as possible (Eckert et al., 2004; Boldy et al., 2011). This lifestyle choice is known as “Aging in Place (AIP).” Assisted living facilities (ALFs) are characterized as a housing-and-services setting to maintain safer, healthier living conditions (Horgas and Abowd, 2004). AIP enables older adults to live in a familiar environment and maintain current social networks and social interactions with close family members Further, their mental functions remain healthy through interactions with their friends in that environment. In addition, a comfortable, familiar environment provides them emotional stability. In the case of ALFs, price may be burden, but tailored services can lead to a more convenient life (Horgas and Abowd, 2004). Providing a safe, secure, and comfortable living environment is important to improve the well-being and happiness of older adults (Perez et al., 2001; Sabia, 2008). Thus, understanding the functions needed to support well-being based on their patterns of living and behavioral characteristics is critical (Costa-Font et al., 2009).

Many studies have emphasized the need to use information and communications technology (ICT) technologies (Deen, 2015), to provide appropriately designed living environment suitable for older adults fitted with embedded sensors and voice-activated services (Ding et al., 2011). The development of ICT helps users to take control over smart technologies in the home (Kerbler, 2016). This idea is generally known as ambient intelligence or the innovative and smart environment. Such an environment combines modern computing, networking, and smart and innovative devices by helping users to communicate with homes and other users through special interfaces in general. Within the context of the smart environment, numerous sensors are connected to the individual's home. These sensors can measure physical and physiological functions and monitor all activities, and they provide the user real time warnings on devices that malfunction. A smart home is an environment that adopts ICT to collect and share information, analyze and monitor residents' behavioral patterns, and improve residents' quality of life (Courtney, 2008; Balta-Ozkan et al., 2013). It is clear that smart technology has a positive effect on overall life because it helps older adults to easily perform essential activities in the space with minimal energy. However, caution needs to be exercised regarding the spaces in which all the functions are automated since they can make older adults frailer by limiting their movements. Deeper consideration is required on the approach to be adopted to automate intelligent systems in such ways that help older adults remain physically and mentally active. A balance needs to be found to provide the right environment without being too intrusive.

Currently, older adults are being exposed to technologies and increasing their experience as regards using interactive technologies with the assistance of the younger population. For information access using technology, a typical human being needs perceptual, motoric, and cognitive capabilities. However, as people age, their cognitive, physical, and sensory capabilities tend to deteriorate (Abegaz, 2014). Frequently mentioned concerns are high cost and privacy implications. Additionally, older adults may believe that smart home technologies are difficult to control or impractical to use (Peek et al., 2014). Participants in pre-implementation studies also expressed concerns regarding the burden it may put on their children in their role as caregivers (i.e., causing workload or worrying) and the possible negative effects on their personal health (Rush et al., 2013; Lee and Coughlin, 2015; Peek et al., 2017). Usually, older adults are afraid of innovation and modern technology; they suffer from so-called technophobia (Sponselee et al., 2007; Booker, 2011) because they are not yet ready to change their mindset toward technology. Older adults who did not grow with current technologies have difficulty accepting smart technologies (Peek et al., 2014). Researchers should carefully examine the ways in which they can help older adults realize the fact that modern technology can help them to be safe within their homes, with independence.

Many studies have been conducted on the smart environment, smart home, and smart technologies designed for older adults or people with disabilities. According to Demiris and Hensel (2008), studies on using smart technologies consider a restricted number of settings based on communities or laboratories, in which the suggested technological innovations demonstrate a high level of feasibility. This study shows that 71% consider technologies for functional monitoring, 67% for safety monitoring, 47% for physiological monitoring, 43% for cognitive support or sensory aids, and 19% for monitoring security, whereas only 19% focus on ways to increase social interaction. The research conducted was directly related to testing and analyzing technological advancements, devices, and sections in the systems of innovative built living environment, that are connected with ensuring control and safety, identifying the activities of users, sending reminders, and evaluating their physiological functions. It should be noted that an insufficient number of studies are related to the analysis of activities and needs of people according to their physical or mental abilities (Cesta et al., 2007; Pecora and Cesta, 2007). One of the needs that older adults have is being independent. Therefore, it is a matter of crucial concern to take into account the perceptions of older adults in terms of smart home technologies since the latter are the tools of improvement needed for quality aging. To provide a pleasurable smart environment, a human-oriented approach should be adopted, rather than a technology-oriented one.

Smart spaces are needed that can provide older adults with pleasurable experiences beyond just usable and practical spaces. We believe that the smallest space design retrofit can have a potentially lifesaving impact. Simple design choices can provide older adults a safer living environment in the same living space to which they are accustomed, and hence enable them to live longer. For this purpose, not only technological interventions but also the roles of design professionals are important. For older adults, a house is not merely a living space. It has diverse sensory and emotional experiences, such as memories, temperatures, smells, and familiarity with spaces. Emotions are a very important part of human life, because they have power to influence the way we make decisions, evaluate risks, solve problems, focus our attention, find something interesting, and categorize information. Such spaces that affect these emotions may make the users want to continuously stay there, and the positive emotions they receive from the space will play a significant role in promoting the well-being of older adults.

## Methodology

### Selection

Research on smart homes and smart environments is being conducted to support well-ness for older adults. Most such studies have focused on support of those over the age of 60, using personalized smart services that enable monitoring and tracking via wearable or implanted sensors. We primarily conducted search through Google Scholar, using terms that included multiple ways of describing older adults (e.g., “older adult”; “senior”; “elder”) and smart homes, such as “smart home,” “smart living,” “assistive,” “smart technology,” “health smart,” “intelligent living,” “intelligent building,” “smart environment,” and “smart technologies to support healthy aging.” We selected papers in the architecture domain only. We examined other sources, including reports, websites, and relevant newspaper articles, to conduct a critical review of smart environments focused on older adults' well-being. A variety of studies on the smart environment for older adults have been published in research disciplines, such as electrical engineering, IT, computer science, gerontology, biomedicine, and robotics. However, we found it relatively difficult to find a substantive amount of formal literature about smart home and smart technology for older adults in the architecture domain. Since the studies on smart environments for older adults published in architectural journals are very scarce, this study included all studies on smart environments even though some did not have older adults as their targets. Among articles on smart environments in the architecture domain, we reviewed 50 publications since 2000, and 30 of these articles were excluded because these are cases focusing on only technical aspects or simulations. This review paper focuses more on design aspects than on technological solutions. Finally, we selected 20 papers for the purpose of identifying factors that should be considered in developing a pleasurable smart environment for older adults.

### Contextual Analysis

We developed a contextual analysis framework for the first stage of the critical review, aiming to extract principal factors that focused on a smart environment to enable pleasurable experience for older adults (Table 1). For each selected paper, we focused on analyzing the following aspects: the demographic considered, the technological features used, the smart environment context focused on, the user experience provided, the methods used in the research, and the ultimate purpose of the research.

**Table 1:** Contextual analysis of smart environment factors from each paper.

	**Dimension**	**Smart features**	**User experience**	**Target**	**Study design**	**Issues (goals)**
Yu et al. (2019)	Living space	Application of unobtrusive sensors	Safe, secure, independent, comfortable, and autonomous	Older adults	Longitudinal pilot study	Understanding the relationship between older adults' daily activities and their living environment
Cho et al. (2013)	Workspace	Functional spaces equipped with smart technologies	Self-development and capacity to work from home	Pre-elderly (40–50 s)	Intensive interview	Understanding the need for smart workspaces for the pre-elderly
Park (2008)	Living space (ubiquitous environment)	Environment behavioral approach	Healthcare, domesticity, mobility and security, network, and recreation	Older adults	Survey	Providing guidelines for a ubiquitous environment (Identify daily activity factors and five affordance dimensions)
Kymäläinen et al. (2017)	Living space (healthcare)	Home control system (actuator; sensors)	Health and safety	Older adults	Empirical study	Proposing a co-design and development process, using persona (Alice)
Chen et al. (2010)	Living space	Living 3.0 Demo prototype (laboratory setting)	Safety, health, sustainability, and convenience	Non-specific	Questionnaire	Facilitating the design of intelligent space based on user acceptance model
Skjølsvold and Ryghaug (2015)	Smart grid	Smart electricity meters	Energy consumption, simplicity, and health	Non-specific	Qualitative study	Understanding social technical aspects of smart gird development
Behr et al. (2010)	Neighborhood	Blueroof technologies (cost-effective wireless monitoring technology)	Innovative, cost-effective, and independent	Older adults	Prototype design	Supporting low-to middle-income seniors to age in place successfully
Dimitrokali et al. (2015)	Living space	Smarter heating control	Energy efficient, cost-effective, education, social network, and better design	Non-specific	Self-report	Understanding homeowners' perceptions and experiences in using a domestic home heating
Kim et al. (2009)	Living space	High-tech amenities	Safety, security, controllability, health, independent, assistive, and autonomous	Older adults	Questionnaire	Investigating user needs on new types of technological systems
Spataru and Gauthier (2014)	Building level	Non-intrusive monitoring system, user location, and tracking	Energy efficiency and comfort	Non-specific	Comparative study	Developing metrics related to total building occupancy and assessing the impact of occupancy on energy use in buildings
Jalal et al. (2013)	Living space	Human activity recognition	Health	Non-specific	Simulation	Proposing novel methodology for recognizing human activity
Hargreaves et al. (2018)	Living space	Smart home services, including energy management, security, and home monitoring	Familiarity, adaptation, training, energy saving, security, convenience, and automation,	Non-specific	In-depth qualitative data; longitudinal study (field trial)	Understanding how householders learn about, use, and adapt to, SHTs in their own homes for energy-saving potential
Mahmood et al. (2008)	Living space	Gerotechnology compensatory mechanism)	Safety, independence, social interaction, use of technology, support, health, and privacy	Older adults	Pilot study	Understanding perceptions (attitude, opinions, and preferences) and use of gerotechnology
Lee et al. (2013b)	Single-person household	Sensors and appliances (smart services)	Convenience, health, efficiency, safety, leisure, and social	Single person	Scenario-based service design	Understanding challenges and suggesting configuration and arrangement method of sensor and appliance for single household
Kim et al. (2017)	Living space	Sensors, devices, and smart appliances	Security, convenience, and connection to others	Non-specific	Scenario-based software architecture	Proposing a holistic and extensible software architecture for heterogeneous smart home systems to enable dynamic integration of devices and services
Barbosa et al. (2016)	Tiny or compact apartment	Smart interior design, use of efficient and flexible furniture, and movable walls	Sustainability, flexibility, and efficiency Energy efficiency, cost, and comfort	Non-specific	Comparative study	Developing smart interior design and space saving techniques to increase land use efficiency of buildings
Darby (2010)	Living space	Smart metering and affordance	Engagement and energy efficiency	Non-specific	Qualitative study	Understanding how householders have used consumption feedback, with and without smart meters and how it can assist with customer engagement
Chien and Wang (2014)	Living space	Smart partition system	Customization and flexibility	Non-specific	Develop prototype; comparative study	Integrating smart technologies into existing buildings
Lee et al. (2013a)	Living space	Smart services based on the spatial behavior pattern of the elderly	Comfort, health, emergency, and convenience	Older adults	Behavioral pattern analysis	Understanding the behavioral needs of the elderly in the bedroom and promoting smart homes to provide support
Park and Kim (2018)	Living space	Voice-activated human–appliance interface systems	Social interaction	Non-specific	Experiment	Understanding natural language commands in smart homes

As a result, providing a healthy, safe, and secure environment and monitoring activities are primary part of smart environment research in the selected paper. Many studies show that saving energy is often an additional benefit for older adults. Whereas, some studies focus exclusively on older adults, the remaining tend to frequently mention older adults because of the characteristics of the studies although they did not specifically target older adults. The proportion of studies using the self-reporting method is higher than that of exploratory studies. Most studies on designing smart environments were conducted on individual spaces; however, a few studies have been extended to the community and environmental level. This evaluation framework was established by gathering all the components considered in selected papers on the smart environment. These components were then categorized into four main categories related to the pleasurable experiences of older adults connected to the smart environment that supports AIP. This analysis is a basic work to extract factors comprising the evaluation framework in section Evaluation framework.

### Evaluation Framework

We developed the evaluation framework by analyzing components of smart environments through the contextual analysis and extracting factors to provide pleasurable experiences in smart environments to enable older adults to live independently and to promote a sense of overall well-being in them (Table 2). The categories for evaluation framework were in divided into four: Well-ness, Independence, Acceptance, and Design. Through the contextual analysis, we found that new technologies make space smarter and advance independence to promote the well-ness of older adults (Vacher et al., 2011; Lattanzio et al., 2014).

**Table 2:** Evaluation framework of smart environment, focusing on pleasurable experience of older adults.

**Dimension**	**Factors**	**Clarification**
Well-ness	Safety	Providing a secure environment to ensure the safety of older adults
	Health	Providing an active environment to promote the physical and emotional health of older adults
	Interaction	Providing an environment enabling older adults to interact with nature, to interconnect with their family in the event of a specific problem or danger, and to participate actively in social activities to avoid being isolated
	Fun (Happy)	Providing an enjoyable environment to enable a variety of activities that allow older adults to identify and pursue their interests and have fun
Independence	Automation	Automating the system for older adults to be able to use the smart environment without extra efforts or ability
	Affordance	Providing clear perceptions of possible interactions between householders and artifacts in smart environments
	Physical support	Supporting older adults who are physically frail to perform activities of daily living
	Cognitive support	Supporting older adults who are cognitively impaired to perform activities of daily living
Acceptance	Positive experience	Considering positive emotions (e.g., satisfaction, fun, and enjoyment) while using smart technology and residing in an independent smart home
	Sustainability	Making the smart environment more sustainable with smart technology
	Perceived usefulness/benefits	Understanding the prospective older adults' perceptions of usefulness, benefits, and risks of smart home environments
	Need finding	Understanding the needs of older adults to provide a pleasurable smart environment
Design	Human-centered approach	Designing a smart environment in consideration of the unique characteristics of older adults
	Individual level	Considering only individual characteristics when designing smart environments
	Community level	Considering smart space in a connected smart community and smart neighborhood when designing smart environments
	Environment level	Considering smart space in a connected smart city, infrastructure, and sustainable city when designing smart environments

The ultimate goal of the smart environment commonly referred to in all the selected papers is to promote the well-being of older adults. We identified the critical factors of a pleasurable smart environments focused on preserving physical, psychological, and environmental well-being. For a pleasurable smart environment, providing a secure, and safe environment to promote the physical and emotional health of older adults is essential. Environmental well-ness involves considering the interaction with not only the natural environment but also the social environment. It is difficult to find smart spaces developed for the purpose of providing fun and interesting experiences to older adults in the papers selected. However, the aspect of fun must be considered to promote emotional well-ness for a pleasurable smart environment.

Older adults strive to maintain their independence and autonomy at the end of life. They prefer to maintain a greater degree of personal independence with help from family, friends, or caregivers. For older adults to control their own lives independently, it is necessary to automate the spaces in which they live (Demiris, 2008, 2009; Helal et al., 2008), particularly when they are too physically frail and too impaired cognitively and hence unable to manage life independently. Automated spaces designed with the idea of affordance can help them to understand the use and function of the smart living environment (Norman, 1999). In the process, it is necessary to support their physical and cognitive functions and to provide usable, affordable options.

The attitude of older adults in adopting smart technologies is also discussed as an important factor for them to enjoy well-being while leading independent lives (Courtney et al., 2008; Pal et al., 2018). Various studies are attempting to understand the characteristics of older adults and apply them to design. To develop a pleasurable smart environment for them, it is important to understand key factors that influence their acceptance of smart technologies. Further, to induce older adults to accept smart technologies, studies must consider factors evoking positive emotions during the use of smart technologies and appliances, providing sustainable smart environments, giving benefit from the use of smart technologies and providing experiences that consider users' needs (Hargreaves and Wilson, 2017).

The aforementioned factors are crucial in considering the design of a future smart residential environment. Designs that demonstrate effective grasp of the characteristics of the elderly can provide pleasurable experiences. The use experience of the elderly in smart homes depends on how the smart home is designed (Eggen et al., 2017). The design should not only meet the needs of the individual using the space but should demonstrate a detailed understanding of the relationships between the individual, community, and environment. The evaluation framework for smart environments for older adults required a critical review of selected papers.

## Critical Review of Smart Environment for Older Adults

### Well-Ness

The overall goal of smart home research is to enable older adults to live independently at home as long as possible for promoting their physical, psychological, and environmental well-ness. In this section, the following four critical aspects for promoting well-ness in smart environments are included: Safety, Health, Interaction, and Fun.

The smart home concept was originally developed with a focus on improving security and energy saving (Chen et al., 2010). In the previous decade, the aim of smart home technologies has gradually expanded to include the purposes of assisting people with disabilities, older adults, and those with reduced capabilities to enrich the living environment, improve comfort and facilitate well-being (Stefanov et al., 2004; Demiris et al., 2009; Ding et al., 2011). Smart homes increase domestic comfort, convenience, security, and leisure as well as reduce energy use through optimized home energy management (Hargreaves et al., 2018). Chen et al. (2010) state that safety, health, sustainability, and convenience are suggested as being the four major goals in the development of an intelligent living space policy. Lee et al. (2013a) analyses the efficiency of a smart service for bedrooms aimed at supporting the older adults' behavioral needs. The focus of the smart service patterns is on emergency, convenience, and health preservation. In particular, one of the needs of older adults is a service associated with emergencies and management of health in the smart home. Kim et al. (2009) studied older adults who need technologies to assist their daily activities at home. The research included ranking of the categories covered by the technological advancements according to their value, and the older adult participants referred to security and safety as the top value. Aging causes a wide range of physical problems and impairments that create an urgent need in new technological systems for older adults that would facilitate safety and maintenance of health under the conditions of the smart environment. In this sense, improving physical health, security, and energy savings are considered important factors when designing a smart environment.

Although these health and safety aspects are important for enriching aging life, happiness is also an essential concept of well-being within the emotional well-ness of older adults. The contribution of technological systems toward improving the quality of life for older adults should focus on social and fun aspects as well. Behr et al. (2010) stated that older adults' active participation in social activities and the establishment of their sense of belonging as a social member have important effects on successful AIP. Home features hardwired with smart communication technology can be used to encourage their social connectedness and prevent those living independently from feeling isolated. In this regard, Mahmood et al. (2008) conducted many pilot studies to find the answers to the questions: How do older adults perceive the use of technology? What factors influence their perceptions about using communication and monitoring technology? According to those studies, smart technologies take into consideration direct communication with friends and family, which facilitates the improvement of health and supports the emotional balance of older adults. Park and Kim (2018) introduced a semi-supervised named entity recognition system for extracting execution targets from natural language commands. They focused on voice-activated human–interface systems in a smart home. They show the possibility that because smart appliances can understand the user's language and react immediately, they can conduct simple conversations with older adults living alone at home. This prevents older adults from feeling alone and isolated. This system can not only enhance the usability of smart technology but also arouse interest in users. Although many studies still emphasize health and safety as the key features of a smart home, we should not overlook the importance of social and fun aspects.

### Independence

Considering automation capabilities within the smart environment to help older adults who are physically frail and cognitively impaired is important to enable them to live independently and to improve their well-being. In this section, the following four critical aspects for supporting their independence in the smart environment are included: Automation, Affordance, Physical support, and Psychological support.

To support the independence of older adults, smart home systems provide automation capabilities that allow them to have control over their living environment and monitor it. According to Yu et al. (2019), in a smart home for older adults, the technologies of unobtrusive sensors can be applied, which facilitate providing health care services and help in evaluation of their daily activities. Thus, it is possible to gather data regarding them with no intrusion into their daily life and routine (Wickramasinghe et al., 2017). This approach can maintain the privacy of older adults in their living space, and their normal everyday life remains the same. Several devices are integrated into smart homes, in particular, for video surveillance, intrusion detection, entertainment, smoke and fire detection, and health monitoring. Many of these devices use different communication protocols with various levels of abstraction that are incompatible with each other (Kim et al., 2017). Kim et al. (2017) proposed an extensible OSGi-based architecture to ensure effective integration of different smart services and devices. These smart sensing technologies demonstrate the possibility of providing physical and psychological support by collecting information about the occupants' behaviors and predicting the behavior patterns in smart environments (Spataru and Gauthier, 2014). Jalal et al. (2013) proposed human activity recognition methodology from recognized body parts of human depth silhouettes to monitor services at smart homes.

Sensing technology and solutions for managing smart devices are required to automate everything in the house and make the living space smart. However, using smart systems can be an extra burden for older adults. It is important to help older people learn to use smart systems independently by informing them about system functions. Many studies (Park, 2008; Darby, 2010; Cho et al., 2013; Maher and Lee, 2017) have considered the idea of affordances to provide a clear perception of possible interactions between householders and artifacts in smart environments. Smart technologies should be designed that allow older adults to communicate with technologies easily, rather than having to learn complex technical languages and commands to support their independent living (Hargreaves et al., 2018). Cho et al. (2013) explained that if the functional and physical environment is well-designed, it provides affordance of smart workplaces, and hence, it is possible to use smart spaces more effectively, since users' understanding of space use increases. Smart appliances presenting in spaces are mapped to behavioral patterns suggested by participants. For example, lamps are automatically turned on, if sensors attached to doors detect that the doors are opened. If smart services are customized to match older adults' behavioral patterns, it is possible to support their physical and psychological independence. The idea of Lee et al. (2013a) is to introduce a smart service on the basis of connecting the behavior of older adults within a space. It should be a service that is customized for a particular smart home and behavior of individual adults and should not be limited to physical assistance alone but should include psychological and social support. Creating and customizing intelligent environment services is important to provide physical and psychological support for older adults.

### Acceptance

Many older adults believe that their independence can be facilitated by their use of smart home technologies, yet these conditions often do not translate into a willingness to accept smart home technology (Courtney et al., 2008; Demiris, 2009). In this section, the following four critical aspects (Positive experience, Sustainability, Perceived usefulness and benefits, and Need finding) are discussed to understand the use and acceptance of smart technology by older adults.

Most previous smart home research has explored the technical challenges of delivering smart domestic environments (Cook, 2012). The majority of this work has not focused on users and their requirements. The recent research demonstrates a growing interest of older adults in devices for smart spaces (Mennicken et al., 2014; Wilson et al., 2015), but determining the aspects older adults like and the solutions suitable for them are issues to be resolved. To design the smart environment in which users may be interested and that they would find acceptable, it is important to properly understand the use and concept of each space constituting a smart house and arrange the smart technologies depending on users' needs. Older adults should evaluate the features and effects of living in a smart environment based on how they perceive and understand it. Cho et al. (2013) identified the key concept and attributes for designing smart workspaces around activities conducted in such spaces based on users' needs and preferences. The approach is unique in that it shifts its focus from the elder people to the pre-elder people. The latter have characteristics that differ from those of older adults. The future development of smart services should be considered by focusing on the needs, technical dispositions, and preferences of pre-elder people. Hargreaves et al. (2018) identified when the users are motivated to use smart technology through in-depth interviews by conducting a longitudinal study. Darby (2010) also pointed out that learning from user experiences is significant in designing a smart environment. According to Lee et al. (2013b), ensuring customization of suitable services for smart homes is complicated because IT developers work on the basis of their own understanding instead of taking into consideration the needs, psychology, and behavior of the actual residents. As a result, residents do not experience the expected level of satisfaction or quality of life on using smart home services. Therefore, performing a need finding process when designing smart environments for older adults is important for providing a satisfactory experience.

According to Chen et al. (2010), users' acceptance appears to be affected by perceived usefulness and perceived enjoyment. Hargreaves et al. (2018) identify that the task of learning how to use smart technologies is demanding and time-consuming. They point out that older adults still do not understand the benefits of smart technologies. No matter how helpful smart systems can be for their daily life, these systems are useless if older adults do not use them. Efforts should be made to make them fully understand the benefits of using these systems. Hargreaves et al. (2018) indicate that a clear understanding of technological and human factors is necessary to design for smart living. In addition, it is difficult to validate the total effect of diverse smart technologies, since smart homes have often been studied in laboratory settings, and hence, they have been rarely applied to the real world. They study to facilitate the design of intelligent space based on an assessment of user needs. It is necessary to study how smart spaces can be accepted by older adults. Chen et al. (2010) introduce the technology acceptance model used to evaluate the complexity and the dynamics of users' perceptions. It emphasizes that perceived enjoyment has positive effects on users' acceptance. Older adults demonstrate a positive attitude toward adapting to new technologies in their residential environment. However, their preferences as regards the control methods for technological systems showed limitations in their adaption to new detailed techniques. One of the desires older adults expressed concerning technological system control was an easy interface that resembles that of television and a remote control pad (Kim et al., 2009). This result showed that the design of technological smart systems for older adults should incorporate easy, user-friendly control without any complicated menus. It is important to provide positive experiences in the smart environment. In this sense, design, and planning considerations could be suggested on the basis of the understanding of older adults.

Smart home devices designed to make homes more sustainable could allow the aging to maintain independence (Skjølsvold and Ryghaug, 2015). Some studies associate architectural design with the choice for materials from sustainable sources, indoor air quality, energy efficiency, and productivity (Barbosa et al., 2016). A smart heating control system is one of the smart devices to which users can have easy access. It is already actively used in many homes because it has the advantages of low price and ease of use. The advantage it offers of saving energy through a simple operation positively affects users, leading them to accept the system without any sense of repulsion (Dimitrokali et al., 2015). Barbosa et al. (2016) studied the application of effective technologies of interior design that can change the living space based on the introduced improvements in environment sustainability combined with the principles of green building. A popular current tendency is using smart interior design with flexible furniture and movable walls in compact apartments. Smart interior design techniques enable saving on building resources and materials, which consequently leads to the reduction in energy required for heating, lighting, and air conditioning. It is possible to transform the living space rather quickly with the use of RoboWalls electric motors that can be controlled with instructions given through a computer interface, smartphone application, or direct voice commands. Such techniques may be an adoptable solution to support independent living for older adults.

### Design

Active research on smart technology and integration of devices into the living environment started as far back as the late 1990s. Regarding the future design and development of smart environments, it is clear that users need to be better accounted for or actively drawn into the design and development process. In this section, the following four critical aspects are included: Human-centered approach, Individual level, Community level, and Environmental level.

The importance of understanding the needs of older adults has been mentioned several times in section acceptance. In addition to understanding these needs, it is necessary to examine the ways in which such understanding can be applied in space when designing a smart environment. Kymäläinen et al. (2017) presents human-centered co-design process with users as an approach for studying intelligent environments. They introduce a variety of design methodologies, such as persona, user scenario, and paper prototypes, to understand usage situation. The design is evaluated through observations, focus group discussion, and interviews. This is the human-centered approach typically used in designing user experiences in the field of human–computer interaction, which can actively reflect needs of users who directly use intelligent spaces, leading to increased acceptance of smart homes. It suggests that it is important to understand users' needs to increase their acceptance. By using the persona called Alice, it introduces a multifaceted design process and challenges, to provide an appropriate design to the persona. Such a design-oriented study should be actively conducted in the field of architecture. Chien and Wang (2014) pointed out that smart technologies should be customized according to user's needs and preferences as part of the human-centered approach. This approach will help to identify how smart technologies are being used and can be integrated into existing buildings in an effective way (Darby, 2010). Adopting this approach is necessary to make users consider the designs and implementation of smart technologies.

The benefits of home automation to a society could be so much more if smart homes were scaled into fully connected smart communities (Behr et al., 2010; Li et al., 2011). Smart city technologies such as smart mobility management tools, smart transportation tools, smart energy grids (Darby, 2010), and etc. can make living environment more effective and efficient. A smart environment should not only help older adults with the activities necessary for life in convenient ways but also provide them with multisensory experiences so that visual, aural, tactile, olfactory, and gustatory senses are stimulated appropriately (Clements-Croome, 2005). The built environment for older adults needs designs that can increase contact with natural light or the external environment. When designing smart environments, it is necessary to pay attention to the activities in cooperation with the community and environment surrounding human beings, rather than focusing simply on the living space used by humans. To understand how to create more productive environments, it is important to understand how humans use spaces. How spaces are designed have a significant effect on air distribution, acoustic quality, natural ventilation, and the amount of daylight (Clements-Croome, 2005), which in turn have a major effect on the fundamental quality of life for humans. In addition, spaces can change a user's patterns of life and behaviors. Various experiences felt in spaces depend on the aesthetic, functional, and emotional properties of the spaces. Cooperation of various fields is essential in developing a smart environment to improve the quality of human life. Living in smart homes has social and economic implications and, therefore, should involve IT specialists, engineers, architects, city planners, and designers covering fields of psychology, sociology, and ethics, and a dialogue and close collaboration should be maintained simultaneously with academia, industry, and policy-makers. Thus, establishing a good-quality scientific experimental platform to conduct interdisciplinary research on issues related to smart homes is essential.

### Summary

Through the critical review of the 20 selected articles, the development of a pleasurable smart environment is complicated and involves multiple factors, such as physical, cognitive, psychological, and environmental factors. The relationships between these factors need to be explored in depth. In this research, we developed an evaluation framework for smart environments to enable AIP. Through the application of this framework, each selected paper in the field of smart environments was evaluated from a balanced perspective for critical review. The results are as shown in Table 3. The Harvey ball was used to interpret the degree to which each factor is mentioned in each paper. In case the ball is filled with black completely, it means that a specific factor is dealt with as an important factor in the paper (Han and Kim, 2018; Lee et al., 2019). Both researchers conducted a thematic analysis to identify major issues while reviewing the selected papers. Since each ball was chosen according to the researcher's subjective opinion, they discussed the elements related to each paper in depth in a debriefing session and finally reached a consensus. A critical analysis of selected smart environment studies identified the types of fields in which studies are being conducted and the fields that require more in-depth studies in the architecture domain. The analysis revealed that studies on smart environments are being increasingly conducted to support the well-ness of older adults. However, studies to support emotional well-ness in a smart environment for AIP are scarce.

**Table 3:** Qualitative critical analysis of twenty selected articles based on the critical evaluation framework.

**Dimension**	**Factors**	**Yu et al. (2019)**	**Cho et al. (2013)**	**Park (2008)**	**Kymäläinen et al. (2017)**	**Chen et al. (2010)**	**Skjølsvold and Ryghaug (2015)**	**Behr et al. (2010)**	**Dimitrokali et al. (2015)**	**Kim et al. (2009)**	**Spataru and Gauthier (2014)**	**Jalal et al. (2013)**	**Hargreaves et al. (2018)**	**Mahmood et al. (2008)**	**Lee et al. (2013b)**	**Kim et al. (2017)**	**Barbosa et al. (2016)**	**Darby (2010)**	**Chien and Wang (2014)**	**Lee et al. (2013a)**	**Park and Kim (2018)**	**Total**
Well-ness	Safety	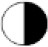	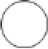	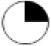	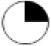	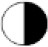	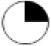	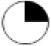	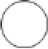	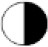	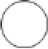	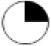	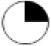	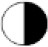	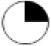	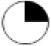	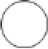	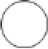	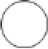	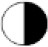	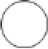	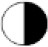
	Health	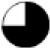	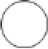	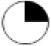	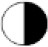	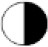	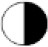	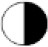	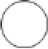	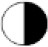	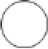	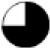	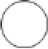	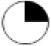	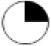	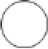	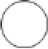	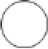	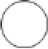	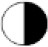	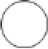	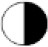
	Interaction	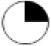	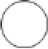	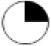	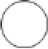	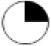	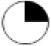	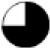	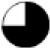	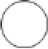	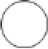	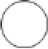	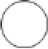	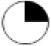	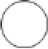	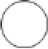	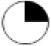	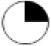	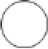	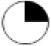	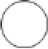	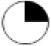
	Fun	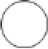	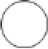	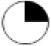	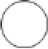	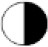	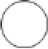	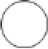	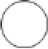	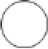	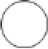	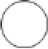	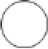	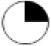	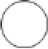	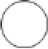	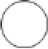	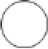	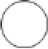	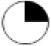	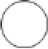	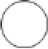
Independence	Automation	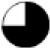	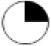	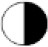	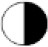	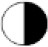	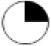	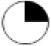	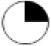	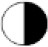	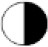	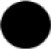	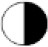	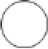	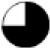	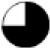	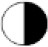	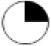	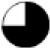	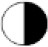	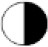	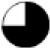
	Affordance	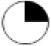	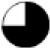	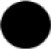	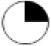	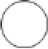	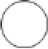	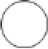	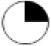	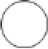	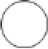	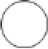	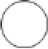	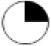	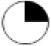	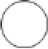	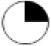	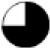	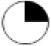	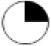	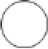	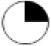
	Physical support	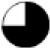	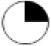	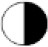	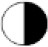	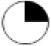	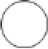	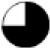	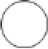	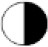	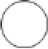	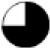	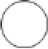	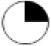	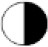	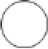	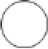	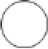	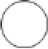	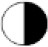	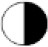	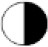
	Cognitive support	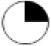	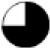	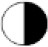	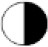	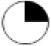	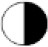	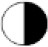	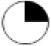	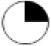	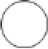	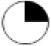	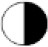	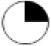	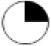	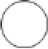	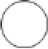	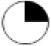	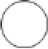	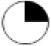	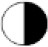	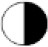
Acceptance	Positive experience	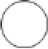	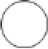	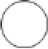	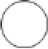	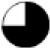	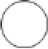	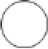	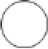	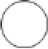	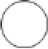	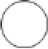	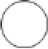	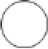	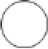	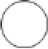	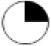	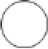	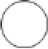	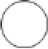	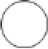	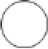
	Sustainability	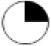	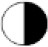	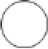	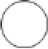	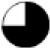	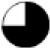	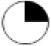	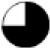	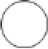	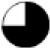	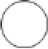	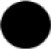	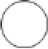	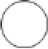	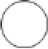	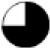	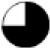	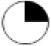	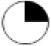	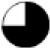	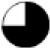
	Perceived usefulness	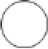	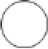	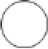	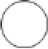	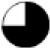	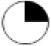	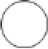	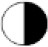	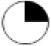	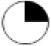	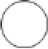	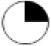	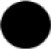	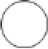	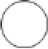	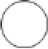	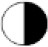	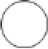	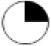	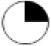	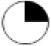
	Need finding	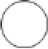	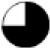	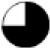	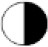	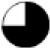	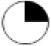	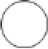	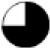	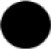	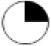	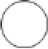	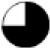	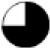	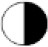	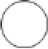	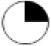	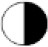	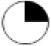	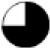	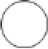	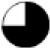
Design	HCA	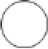	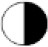	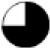	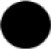	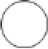	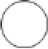	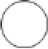	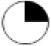	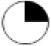	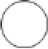	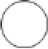	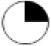	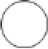	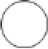	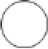	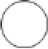	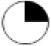	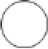	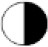	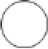	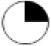
	Individual level	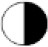	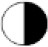	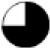	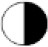	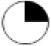	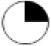	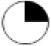	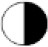	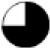	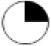	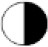	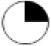	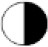	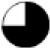	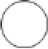	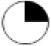	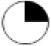	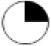	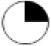	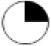	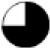
	Community level	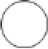	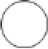	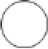	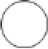	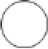	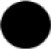	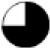	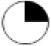	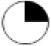	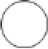	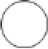	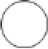	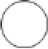	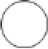	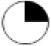	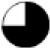	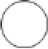	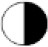	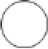	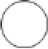	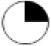
	Environment level	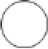	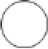	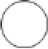	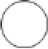	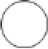	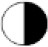	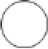	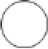	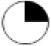	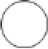	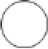	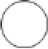	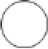	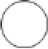	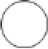	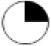	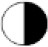	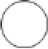	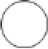	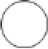	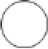

*Harvey balls 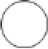

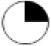

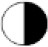

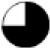

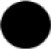
 represent “very poor,” “poor,” “average,” “good,” and “very good,” respectively*.

Relatively many studies have been conducted to understand the characteristics of the manner in which older adults lead their daily life within a space. Several studies considered ways to integrate various sensors effectively into their living space for automation. Many studies refer to the importance of protecting and supporting the health and safety of older adults but rarely mention ways to support methods of improving positive psychological states. Because studies on a smart environment often aim to facilitate the physical independence of older adults in their living space, it was confirmed that factors related to independence are mentioned relatively evenly in the selected papers. It was also found that these studies understand the importance of understanding the personal tendencies and characteristics of older adults and applying them to the design when designing a smart environment but lack in researching how smart spaces can be expanded and connected to a smart community and smart city. Table 3 shows that many studies are missing the need for space intended for the emotional satisfaction of older adults. These missing factors can be the necessary future direction of smart environment research for older adults to realize the vision of AIP to support pleasurable experiences.

## Challenges and Design Issues of Pleasurable Smart Environments for Older Adults

We identified a future research direction through the critical review using the evaluation framework. The selected papers were reviewed on the well-ness, independence, acceptance, and design aspects. In this process, we identified the challenges and design issues that arise in providing a pleasurable smart environment for older adults.

### Smart Environment as a Friend to Promote Emotional Well-Ness

Under the Well-ness dimension, many studies tend to design a smart environment to monitor the behavior of older adults, to evaluate their health status, or to protect them from the risks occurring in the living space. Unobtrusive sensors are installed in various places to monitor the living environment. The strength of the smart environment is that it automatically tracks older adults' behaviors and provides the information necessary for them (Kim et al., 2013). However, it requires many sensors and cameras for detecting their behaviors and location continuously to provide this function. We would emphasize the importance of smart systems that can mitigate the emotional loss experienced by older adults, and not smart systems that only support their functional status. Smart appliances or sensors can be utilized to further reveal design factors in the space and encourage older adults to interact with living environments to provide them hedonic and pleasurable experiences. Aging adults can be affected by their living environment. For example, Amazon Echo and Alexa have shown in many studies the possibility of actively incorporating voice-activated sensors in the lives of older adults, including such cases as inducing them to listen to music or to control the thermostat and reminding them of important events. Such sensors can be integrated into a space to induce or initiate certain behaviors of these adults (Forster and Van Walraven, 2007; Miller et al., 2011, 2014).

In addition, many papers deal with health and safety as an important issue, whereas few studies consider social or entertaining factors for older adults in the smart environment. Many other research disciplines pointed out that active participation and engagement in social activities are critical for maintaining a good quality of life for older adults. It is important to consider the ways in which the smart environment should be designed to promote the emotional well-ness of older adults. The quality of life of an individual is increasingly influenced by interconnections with others (Hirsch et al., 2000). For this reason, several technologies, and robotics have been developed to enhance communication between older adults and their families, relatives, friends, nurses, and doctors to support social interaction. Wang et al. (2014) indicate that aging adults overcome loneliness, anxiety, and depression when communicating with companions. The failure of developers of new and innovative technologies to put into consideration the emotional and social aspects of older adults in their technologies can be a cause for many missed opportunities. A smart space does not stop at facilitating smooth communication with others, and the space itself can play the role of a friend. The conversational agent in the smart living environment can promote social relationships for older adults, acting as a friend or family, not simply as a machine. Since older adults regard human relationships as important, the smart environment should be designed to simulate or stimulate the role of a friend or family.

### Smart Environment to Support Emotional Independence

The selected papers for this review paper present that smart homes and environments can be an option to assist older adults to live independently and maintain an acceptable quality of life (Jacelon and Hanson, 2013). Through the review process, we found that many studies focus on investigating ways for supporting the independent life of older adults. Nevertheless, many studies tend to focus on designing an assisted environment to support the basic life and physical functions of older adults, but often exclude their cognitive or psychological aspects (Mann and Milton, 2005; Hong et al., 2012). The smart environment for older adults should be designed so that it can allow them to lead a satisfying life in the space by monitoring their behaviors and monitoring their emotions. Understanding the relationships between human emotions and interactive systems would have a positive impact on social, cognitive, physical, and other human behaviors. The integration of affective computing and intelligent interfaces is one big opportunity for proving them emotional support (Kuderna-Iulian et al., 2009; Luneski et al., 2010). More improvements of the smart environment for older adults will be realized with the interpretation of sensor data on their emotional status by using affective computing that considers facial expressions, gestures, or speech output of the users (Tao and Tan, 2005).

The smart space that can detect emotional changes in older adults becomes able to provide them personalized information. A personalized adaptive space can be defined as an intelligent space in which the space learns patterns of usage for each individual user and adapts its behavior to that person in a non-trivial way (Jameson, 2008; Surie et al., 2013; Schmidt and Braunger, 2018). The smart space can satisfy users' needs autonomously by recognizing and inferring their behavioral and emotional patterns and can make decisions for older adults when appropriate. They gradually show a passive attitude in overall life owing to physical and mental constraints. The smart environment for them should play a role in drawing their positive emotions. The smart environment should be designed to provide proactive feedback to them generically instead of waiting for their responses. The smart technologies should recognize discomfort so that the user does not feel negative emotions when living in the smart environment. Providing proactive feedback helps older adults know factors to which they should pay attention, and such feedback includes suggestions and helps them make decisions.

### Challenges to Understanding the Needs of Older Adults Related to Smart Environments

Smart homes and the smart environment should be introduced to older adults with careful considerations of their strengths and potential risks. Financial accessibility or affordability should be considered. In the environment in which older adults reside themselves, a small risk may come up as a big problem. Older adults are less likely to feel the necessity of building smart systems by investing a large amount of money owing to ambiguous fears about whether they will use such systems for a sufficiently long period that justifies such expense or about whether they will be able to use these systems properly (Gunge and Yalagi, 2016). To understand their needs and provide a smart environment appropriate for them, it is necessary to understand them correctly (Haines et al., 2007). They face additional challenges because as people grow older, their cognitive, physical, and sensory abilities change, causing older adults to show different attitudes toward technology. Researchers easily overlook that these adults have heterogeneous characteristics (Courtney et al., 2008). They introduce elements such as bigger prompts, high contrast, and simplified interactive functions as design solutions for older adults. These factors could assist older adults to have smooth interaction, but also could reduce their interest. Currently, they are being exposed to technologies and increasing their experiences with the assistance of the younger population. It is increasingly important to understand when, how, and why older adults are engaged.

Technical and psychological accessibility can be addressed by fully investigating the views and needs of older people when implementing smart homes (Lê et al., 2012). Nonetheless, another problem related to implementing the smart environment for them is that very few empirical experiments have been conducted on this issue (Brush et al., 2011). Many studies still depend on the self-report methodology to ascertain older adults' evaluation of the smart environment. The main challenge of self-report methods is that these rely on users' recollection and self-interpretations. People tend to provide responses that are more positive or more frequent than in reality. It is necessary to investigate the problems that can occur while older adults are residing in the smart space themselves.

### Role of Architecture Domain in Designing Smart Environments

Various smart technologies are continually evolving. Architecture or housing is the basic space for human life. Smart homes should prioritize a comfortable and pleasant space for people to live, instead of aiming at housing where smart technologies are installed. People's lifestyles are constantly changing, and accordingly, the buildings in which they reside, are also changing. In addition, smart technologies in the buildings should be changed according to changes in people's lifestyles and housing structures. The smart environment should be able to support the interaction between people and space. It is necessary to conduct research concerning the skills needed for older adults and the design of a pleasant space for them to live (Labonnote and Høyland, 2017).

People in the past usually slept in the bedroom, watched TV in the living room, and worked in the study. However, thanks to the ICT devices and smart technologies that allows the overcoming of space restrictions, people can work in bed and watch TV, and even search for news in the bathroom. With the introduction of these smart devices, people are living in the age in which the boundary of space disappears. To have older adults adapt to smart environments surrounded by technology, they should not be required to engage in a complicated process for using that technology. Thus, in the architecture field, it would be necessary to conduct a study to propose ways for the design of a new space for older adults (Lê et al., 2012).

## Conclusion

Our society will change, and older adults will increase. They will inevitably be surrounded by smart services and technologies in their living environment, regardless of their wishes in this regard. Given that the proportion of older people is increasing more rapidly than that of the younger generations, research related to aging, and smart technology will be needed in various fields in preparation to meet the needs of this aging population. Many studies are developing technologies and environments necessary for extending the lives of older people. Through this analysis, we realized the need for smart technologies that will allow older adults to live well, rather than technologies that will prolong their life. The four dimensions we proposed are widely applicable to the smart environment research on older adults. The main findings derived through this framework are as follows:

1. The main goal of smart technologies is to enable older adults to maximize their safety, strength, balance, fitness, independence, and mobility as they age. The use of smart technologies to support physical conditions and mental health of these individuals has increasingly attracted the interest of researchers across the computing and design disciplines. However, many studies are still focused on promoting the physical independence of older adults and ignore their psychological independence.
2. Independence of the elderly is emphasized in the selected papers. However, these overlook the fact that the positive mindset of older people has a positive impact on their physical and mental health. Therefore, it is necessary for future studies to examine the psychological satisfaction that is required to facilitate their independent living. A smart environment that simultaneously provides a pleasurable experience and assists the physical, cognitive, and psychological activities of older adults is important in enabling them to lead a satisfactory life in old age.
3. Many papers present the importance of understanding the characteristics and attitudes of older people. However, many smart living environments are still designed based on a shallow understanding of them. Hence, since it is important to understand key factors that influence their acceptance of smart technologies, researchers must strive to develop an evaluation framework, or principles that can be commonly applied to evaluating the emotional needs and engagement of older adults.
4. A space with a well-designed smart system can trigger certain behaviors of older people. Therefore, studies need to be conducted on smart environments that can induce active behaviors of these individuals, since it would allow us to understand interrelationships between technology and design and develop ways of bridging the knowledge gap between diverse disciplines.

Because this study reviewed a selected list of journal articles published in the field of architectural studies, one limitation is that it excluded the views on smart environments covered in other research fields. It should be noted that it is difficult for this study to address an integrated, synthesized overview of the current state of smart residential environments for older adults since it did not conduct a systematic review through meta-analysis. However, this study would provide readers the perspectives on the current status of research related to the smart environment in the architecture domain. We identified research challenges and design issues through a critical review of selected papers with the objective of enabling the well-being of older adults through a pleasurable smart experience. The details covered in the evaluation framework are critical factors that should be considered in providing this type of environment. These details can be widely used across stages, from the beginning stage of understanding the target user to the design stage and the system evaluation stage.

## Data Availability Statement

All datasets generated for this study are included in the article/supplementary material.

## Author Contributions

LL composed this study, designed the framework, and completed the quantitative analysis. MK provided supervision throughout the research and contributed substantially to the analytical part of the research.

### Conflict of Interest

The authors declare that the research was conducted in the absence of any commercial or financial relationships that could be construed as a potential conflict of interest.
